# Moral Hygiene

**Published:** 1878-12

**Authors:** 


### MORAL HYGIENE

Is the relation between the state of the mind and the condition of the body. There are some who can say things which "hurt the feelings" of others with perfect indifference, without any compunctions whatever; these have rude natures always, and are just as low bred as a certain kindred class who seem to pride themselves on their "bluntness," which they are wont to dignify by the term of "frankness;" a proper analysis of such minds will show that they are wanting in the finer feelings of our common nature and in that delicacy and refinement which makes all the difference between the courtier and the clown. A gentleman or a lady may inadvertently "hurt the feelings" of another, but to do so deliberately, is as impossible for them, as it would be for a christian to commit a sin intentionally. It may not seem much to wound another's feelings, but who does not know that both men and women have been "mortified to death" literally; have committed suicide while laboring under the influence of wounded sensibilities. Many a delicate nature has been pained to the quick by being passed on the street, without recognition from a friend or an acquaintance, especially if superior in social position. Many a worthy heart has pined in oppressive sadness for weary weeks, at not having been invited to a party given by some associate. One of these "woundings" may not be much, but repetitions may be ruinous. One slight scratch of a pin may be a trifling matter, but its repetition in the same spot will soon induce fearful convulsions ; a drop of water on the head from the height of a yard or two is not much, but if repeated for a time, it is said to induce insanity. There are at all times persons pining away into the grave from the influence of mental states, as remorse, wounded vanity, mortified pride and misplaced affection. If then the preservation of life, and the maintenance and promotion of health are duties incumbent on all, as none will deny, then do we owe it to ourselves, to our neighbors, friends and kindred, to do all that is practicable to promote in one another pleasant, agreeable and profitable states of mind ; and on the other hand to avoid scrupulously and studiously doing or saying anything which would wrongfully, uselessly or unjustly cause an unpleasant frame of mind in another, and thus, in the expressive phrase of Holy Scripture, be " void of offence."

